# Why Is It So Hard to Evaluate Knowledge Exchange? Comment on "Sustaining Knowledge Translation Practices: A Critical Interpretive Synthesis"

**DOI:** 10.34172/ijhpm.2023.7549

**Published:** 2023-06-27

**Authors:** Kathryn Oliver

**Affiliations:** Department of Health Services Research and Policy, Faculty of Public Health and Policy, London School of Hygiene and Tropical Medicine, London, UK

**Keywords:** Evaluation, Interdisciplinarity, Boundaries, Knowledge Exchange

## Abstract

Despite a growth in knowledge translation (KT) or exchange activities, and a smaller growth in their evaluations, it remains challenging to identify evidence of efficacy. This could be due to well-documented political and logistical difficulties involved in evaluating knowledge exchange interventions. By bringing in theory from science and technology studies (STS), Borst et al^1^ offer a new way of thinking about this problem. Most KT evaluations draw on health research traditions; centralising comparability, efficacy, and so on. Borst et al propose focusing on the work it takes to move knowledge over boundaries between these communities, seeing relationships as interactions, not just conduits for evidence. They show how ‘context’ can be understood as a mutual creation, not a static environment; and that institutions shape behaviours, rather than merely being sites or platforms for evidence mobilisation. Seeing KT as a creative, active practice opens new ways to design and evaluate KT mechanisms.

 There is a strange paradox within the world of research into evidence production and use. On the one hand, we observe that lessons about how to make, mobilise and use evidence travel well across disciplinary and sectoral boundaries.^2^ Yet on the other, it is almost impossible to find any concrete, robust evidence about how to mobilise evidence effectively.^3^ What explains this disconnect? How can we be learning so much – yet unable to say clearly “what works”?

 A partial answer might focus on the challenges of evaluating knowledge translation (KT) interventions. Frequently, KT interventions are done as part of a research project, tacked onto the end to do some dissemination and knowledge exchange.^4^ In these cases researchers, understandably very wedded to their projects, can focus on publicising their results, rather than on learning about knowledge exchange *per se.*^5^ Evaluations, where done at all, can consist merely of a brief survey about whether participants enjoyed themselves. Although the evidence about knowledge exchange suggests that relational work, from shared problem-framing through to implementation is most likely to support social change, most funders, with some honourable exceptions, persist in funding ‘impact’ as an add-on, usually to projects they are also particularly attached to. Politically, therefore, it is very difficult for any evaluator, internal or external, to draw out learning about the costs and benefits of knowledge exchange. Logistically too, this add-on model often means that researchers have moved on and there are limited opportunities for people to develop skills, let alone careers in knowledge mobilisation as a practitioner and/or scholar, or a community of practice around knowledge mobilisation activities.^6^

 Of course, there are examples where research into knowledge mobilisation and exchange has been conducted independently of other research projects. These can often involve building capacity in a cohort of individuals, to develop skills in – for instance – knowledge brokerage.^7^ Should an evaluation find no, or negative effects, the implication would be that this cohort of people should lose their jobs. Again, politically and logistically, a tricky evaluation to conduct. These challenges perhaps explain why, more often than not, KT interventions and activities are not evaluated.^3^

 This scatter-gun approach to knowledge exchange also speaks to a more serious theoretical vacuum. These activities rarely articulate their goals or outcomes (or certainly not in much more detail than ‘influencing policy’). As an illustrative example, the terms knowledge mobilisation, exchange, transfer, translation and use are used almost interchangeably, despite having different aetiologies and even ontologies. ‘Transfer’ for example, is wedded to the idea of the linear, problem solving model, which implies that more dissemination is all that is required to cause a change in decision-making. ‘Exchange’ is tied to a more relational ontology which implies mutual learning and adaptation between research users and producers.

 Without a clear understanding of what activities can realistically achieve, or which activities are likely to generate different outcomes, interventions are very unlikely to contribute to the evidence base about KT. Similarly, most are launched into without any attempt to identify similar complementary, competing, or other ongoing processes and interventions which might interact with the new kid on the block. As funders and universities persist in focusing on the marketing of projects and individuals, rather than on the evidence-policy system, the increasingly busy and chaotic mass of activity makes it almost impossible to attribute any effect. Without a clear theory of change, with no systems perspective – interventions will not generate useful learning.

 And this points us to a second possible answer as to why evaluations are so rare; what does ‘good’ look like in this space anyway? Is ‘What Works?’ a useful question to ask? As Borst et al point out, many studies focus on organisational, structural or procedural aspects of KT initiatives. For example, evaluations of KT architecture like the Collaborations for Leadership in Applied Health Research and Care note that different models evolve and appear to support knowledge exchange.^8,9^ Yet, these mostly conclude that the models are very context dependent, not least in terms of the local funding and political environments and interpersonal networks within which the initiatives are embedded.

 The evaluation framing most commonly used to think about how KT works is the health-derived set of questions about what changes, for whom, how and under what circumstances.^10-12^ Good KT evaluations exhaustively document contextual details – but given the heterogeneity between settings and contexts, this evaluation frame makes it almost impossible to derive what health sciences would consider to be ‘empirically robust’ evidence^13^; ie, robust enough to make recommendations for ‘best practice.’ It could be that KT activities simply are not amenable to ‘standard’ evaluation.

 Borst et al^14^ offer a potential explanation as to why this might be the case. They do this by bringing insights together from across disciplines and sectors. They explain the intellectual tradition behind the three conceptual elements of translation from science and technology studies (STS); ie,

 “*[bringing] something from the world into somewhat secluded and protected research spaces – think of blood samples or population data. … [then secondly] the research space is made to resemble the outside world as much as reasonably possible, but is at the same time meant to protect against distortion from the outside world… In the third translation, the researchers may aim to ‘implement’ their knowledge into existing practices…[which]…need to change and the conditions under which the knowledge was produced in the research space need to be reproduced in the utilisation environment as well”* (p. 5).

 This to-ing and fro-ing between the ‘research’ and ‘real world’ spaces shows how STS scholars conceive of different communities of practice, which are reinforced by the objects moving across these boundaries. This, they contrast with how health sciences understand translation, which is less to do with how people interact with each other and with objects, and how these interactions create social spaces, and more to do with the process of knowledge production. Traditional conceptions of knowledge mobilisation in health have drawn on a linear pipeline model, or latterly a linkage-and-exchange model, which also emphasises the importance of relationships.^15^ The contribution the STS theory can bring here, as described by Borst et al, is the theorisation of those relationships, from an informal but necessary ingredient, to the very site of knowledge creation, understanding and action.

 The authors offer further STS-derived insights for health science scholars; that knowledge is always socially situated, meaning that no evidence is ever neutral or objective – it can never stand alone and speak to all audiences. Knowledge will always have baggage, a history, which needs to be understood for it to have meaning for people. For example, when the first X-rays were seen, it was not immediately obvious that they were representations of internal structures – that had to be learned, and meaning attached. Context, which in health is often understood as ‘local environment and setting,’ is used in STS as a way of defining a boundary around an object of study. This implies that creating context is not a static aspect of KT, but an active part of the work done by those involved in mobilising knowledge. Finally, they describe the static version of institutions found in the health literature; with rules, structures and organisational cultures which create a stable, resourced environment to support evidence production and use. The STS perspective on institutions focuses on how these bodies operationalise power; rather than supporting evidence use, institutions rather determine how evidence is made and used, through governing behaviours, not merely facilitating them.

 The authors take these insights and apply them to understanding KT. They way they do this offers important implications for those of us seeking to research evidence production and use. Rather than focusing on diagnosing success and failure, Borst et al focus on what it takes to make this type of activity work. They raise the question of sustainability as an empirical research topic, asking what would it take to make these KT initiatives work in the long term. As with the literature on barriers and facilitators of evidence use, existing work seems to have produced lists of factors, which may or may not be essential for KT work, rather than actionable knowledge about how to make KT work in reality. Borst et al focus on sustainability, but their approach is more widely applicable to many of the knotty problems in this field – learning about what it takes to engage, rather than list a set of conditions which lead to ‘engagement’ seems likely to bear juicier fruit.

 For evaluators of KT, the implications are clear. Learning about how knowledge mobilisation happens in practice, how those involve understand and create their social environments, and how these behaviours and perspectives are shaped by the institutions they exist within, is a difficult, but clear task for evaluations in the future. Borst et al show how the work people do, the practices they engage in, is the front and central task to understand in knowledge mobilisation. Unpacking how people’s behaviours shape and are shaped by institutions; how they legitimise and grant authority to certain forms of knowledge; how daily activities and interactions constitute *work – *all this helps evaluators to formulate new approaches and new questions which might generate useful knowledge. This approach also fits with what we know about knowledge exchange, which suggests that initiatives function most effectively where they are embedded within a science/policy system and connected with ongoing structures and processes,^3^ not organised on a per project basis.

 Much of the work in this field which has sought to learn and synthesise across settings and projects has drawn on intervention and evaluationist perspectives – seeking to learn what works for whom, under what circumstances. It is refreshing to have a different learning route marked out, focusing on what we do (sustaining, in this case), rather than the effects of what we do (sustainability, as an outcome).

 Can we make knowledge exchange easier to evaluate? In my view, the answer has two parts.

 Firstly, for those of us designing, funding and implementing KT activities (also dubbed ‘knowledge mobilisation, academic-policy engagement, research uptake’ and so on), there are some clear implications:

- Interventions should have a clear theory of change. Where the goals are articulated, it is easier to shape activities and mechanisms to reach these goals.
- Think about the mode of action, or mechanisms which will generate these outcomes. Borst and colleagues’ paper offer some ways to identify these proximal and distal outcomes, by highlighting work, practices, and everyday elements which constitute legitimate targets of study.
- Take a systems perspective. What are the complementary, competing initiatives, processes and structures also going on? What can be built on? What needs to be removed? What will this initiative disrupt or add?

Secondly, we can all benefit from taking a more interdisciplinary perspective in this field. Here, the authors help us to think about generalisable practices, embodied knowledge and expertise. Borst and colleagues’ paper is an example of how to move forward sticky problems, learning across boundaries, translating between people and places. Bringing together insights from different disciplinary fields is a very fruitful strategy in this field (see, eg, Greenhalgh’s work on diffusion of innovations), and one which is needed to improve the theoretical and practical knowledge in our field.^16^ To make, mobilise and use evidence, we need to do more of that.

## Acknowledgements

 With many thanks to the many participants and collaborators in knowledge exchange activities I have joined, run or evaluated, and to the many colleagues who have helped me to understand these challenges. All mistakes and opinions are mine.

## Ethical issues

 Not applicable.

## Competing interests

 Author declares that she has no competing interests.
